# RNA G-quadruplex-protein interactions: from nuclear RNA processing to cytoplasmic stress response and neurodegeneration

**DOI:** 10.1080/15476286.2026.2658922

**Published:** 2026-04-10

**Authors:** Dimitrios G. Anastasakis, Markus Hafner

**Affiliations:** a Department of Basic Sciences, School of Medicine, University of Crete, Crete, Greece; b RNA Molecular Biology Laboratory, National Institute of Arthritis and Musculoskeletal and Skin Diseases, National Institutes of Health, Bethesda, MD, USA

**Keywords:** RNA G-quadruplex, RNA-binding proteins, liquid-liquid phase separation, stress granules, neurodegeneration, ALS, co-transcriptional splicing, rG4 homoeostasis

## Abstract

RNA G-quadruplexes (rG4s) are stable secondary structures formed by non-canonical Hoogsteen base-pairing of guanine-rich sequences in precursor and mature messenger and non-coding RNAs. We review evidence that rG4s exist in two functionally distinct worlds. In the nucleus, rG4s fold co-transcriptionally to regulate gene expression and RNA processing and organizing membraneless organelles through liquid-liquid phase separation. Splicing regulation by rG4s is restricted to vertebrates and co-evolved with transcriptome complexity. In the cytoplasm, rG4s are actively maintained in an unfolded state by dedicated helicases and RNA-binding proteins, but fold upon stress to nucleate stress granules, that sequester mRNAs and sustain cell survival. When compartmentalization of rG4-protein interactions fails, cells lose both nuclear RNA processing control and cytoplasmic translational regulation and proper stress response. The same biophysical properties that make rG4s effective scaffolds for reversible phase separation in RNA processing, proteostasis, and acute stress become liabilities under chronic conditions: in ageing neurons, failure of rG4-protein homoeostasis transforms protective condensates into irreversible aggregates associated with α-synuclein, tau, TDP-43, and FUS pathology. We discuss the implications of a dynamic equilibrium of folded and unfolded rG4s in health and disease, with particular focus on their emerging roles in neurodegeneration.

## Structural and biophysical properties of rG4s

**Figure 1.**
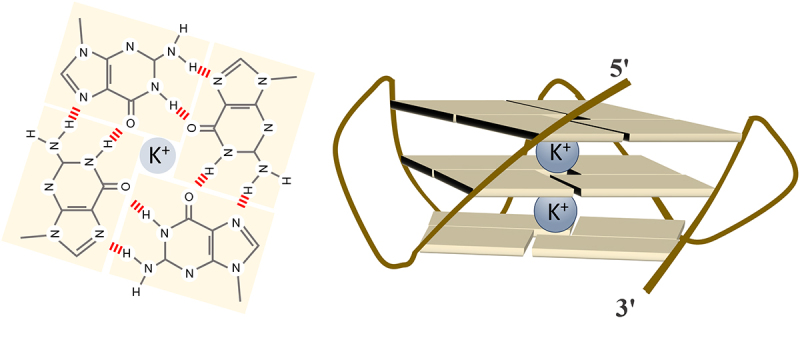
Left Panel: A planar G-quartet formed by Hoogsteen hydrogen bonds between four guanine bases. Hydrogen bonds are indicated by dashed lines. Right panel: Parallel G-quadruplex topology showing three stacked G-quartet layers. Potassium ions (K^+^, grey spheres) coordinate.

RNA G-quadruplexes are four-stranded secondary structures formed when guanine-rich sequences fold into stable helical conformations through Hoogsteen base pairing. Hoogsteen base pairing in G-quartets creates a central cavity that precisely accommodates monovalent cations positioned between stacked quartet layers [1]. This cation-dependent stabilization mechanism provides a unique structural characteristic that fundamentally distinguishes G4S from canonical Watson-Crick helical structures. Watson-Crick base pairing relies solely on hydrogen bonding between complementary bases and does not require centrally coordinated cations within the base-paired structure itself [2]. The canonical G4 motif in both DNA and RNA consists of four stretches of two or more consecutive guanines separated by one to seven nucleotides (G_2+_N_1+__7_G_2+_N_1__7+_G_2+_N_1+__7+_G_2+_), where the guanine quartets are stabilized by π-π stacking and coordination with monovalent cations, particularly potassium [1,3–5]. Cation-dependence establishes a minimum stability threshold– at least two stacked G-quartet layers are necessary to coordinate a single stabilizing cation, while structures containing three or more G-quartet layers achieve significantly enhanced stability through coordination of multiple cations positioned between successive quartet planes, dependent on ionic environment (Figure 1). K^+^ provides optimal stabilization, fitting ideally between the quartet planes to coordinate carbonyl oxygens, while other monovalent cations provide reduced stability (K^+^ > Rb^+^ > NH_4_^+^ > Na^+^ > Li^+^) [6,7].

G-rich sequences were shown to possess gel-like properties much earlier than the discovery of the quartet structure itself [8]. These properties are now linked to the propensity of G4 structures to undergo higher-order self-assembly and biomolecular phase separation, phenomena that have gained renewed attention in recent years. rG4-containing RNAs can undergo liquid-liquid phase separation independently, as demonstrated by the SHORT ROOT (SHR) RNA in plant cells, where rG4 structures alone trigger phase separation under physiological conditions [9]. The capacity of rG4 structures to drive phase separation appears to be an intrinsic property of the G4 fold itself: minimal *in vitro* systems composed solely of rG4-forming oligonucleotides and arginine-glycine-rich (RGG) peptides undergo phase separation in a G4 fold-dependent manner [10].

RNA G-quadruplexes (rG4s) differ from DNA G-quadruplexes (dG4s) in several key structural and functional aspects. Most notably, rG4s are more compact and stable than their DNA counterparts due to the 2′-hydroxyl group in the ribose sugar and appear to prefer all-parallel topologies [11,12]. Several comprehensive reviews have synthesized our current understanding of rG4 biology, covering their structural properties, genomic distribution, biological functions, and computational prediction methods [13–16].

## Computational and experimental detection of rG4s

The study of rG4s relies on computational and experimental approaches, each with distinct advantages and limitations. Computational prediction algorithms such as G4Hunter, QGRS Mapper, rG4detector, and G4RNA screener identify potential G4-forming sequences based on sequence composition, thermodynamic parameters, and machine learning models [17–20].

However, sequence-based predictions often overestimate the number of functional rG4s, as the majority of RNA sequences with G4-forming potential remain unfolded in cellular environments, as determined by DMS-based chemical probing in living cells [21]. This global unfolding, maintained by helicases and RNA-binding proteins that actively resolve rG4 structures, raises the fundamental question of which rG4s do fold *in vivo* and under what conditions. An alternative chemical approach using N3-kethoxal, has also been applied to probe rG4 formation in live cells with limited coverage, potentially due to insufficient sequencing depth or the inability to capture highly dynamic rG4s [22]. Pull-down approaches using rG4-specific ligands or antibodies combined with RNA sequencing provide transcriptome-wide identification of endogenous rG4-containing RNAs, but require careful evaluation for background interactions and for the dynamic folding and unfolding of rG4s, which can occur post-lysis in cells [15,23]. G4-RNA-specific precipitation followed by sequencing (G4RP-seq) uses formaldehyde crosslinking to capture snapshots of transiently folded G4 structures across the transcriptome, followed by affinity purification with G4-specific small molecule probes [24,25]. This approach detected widespread transient G4-RNA formation in cellular RNA populations, suggesting that rG4 structures continuously form and are rapidly resolved, reconciling observations of rG4 structural stability *in vitro* with their predominantly unfolded state *in vivo*. Strikingly, transcriptome-wide chemical probing (SHALiPE-seq) demonstrated that hundreds of rG4s are stably folded in *vivo in* both Arabidopsis and rice, providing direct evidence of rG4 formation in living eukaryotic cells [26]. Recent methodological advances have improved both sensitivity and specificity: rG4-seq 2.0 achieves comparable detection with as little as 10 ng of input RNA, enabling analysis of low-abundance samples [27], while d-rG4-seq applies DMS-based probing specifically to chromatin-associated RNA, revealing that ~1% of the 5,000–6,000 *in vitro* rG4s were detected *in vivo*, but this may be an underestimate [28]. More recently, ultra-low-input rG4-seq (ULI – rG4-seq) enables transcriptome-wide rG4 detection with ~140 bp resolution from as few as 100 cells, revealing rG4 enrichment near transcription start and end sites [29]. *In vitro* transcriptome-wide experimental studies reveal approximately 3,000–13,000 rG4-forming sites in polyA-selected RNA from human cells, depending on experimental conditions, with rG4-seq detecting 3,845 sites under physiological K+ concentrations and 13,423 under G4-stabilizing conditions in HeLa cells (Table 1) [23,30].

**Table 1.** Transcriptome-wide methods for RNA G-quadruplex detection. Summary of experimental approaches developed for transcriptome-wide identification and characterization of RNA G-quadruplex structures. Listed are the method name and underlying principle, RNA substrate, input requirement, experimental context (in vitro or in vivo), number of rG4 structures or transcripts detected, and the biological system used. RT-stop, reverse transcriptase stalling; NAI, 2-methylnicotinic acid imidazolide; DMS, dimethyl sulphate; BioTASQ, biotinylated G-quadruplex ligand; PDS, pyridostatin; cPDS, clickable pyridostatin; poly(A), polyadenylated RNA; mESC, mouse embryonic stem cells; HEK293T, human embryonic kidney cells; K^+^, potassium ions; Li^+^, lithium ions.

Study	Method	RNA type	RNA input	Condition	rG4s detected
[30]	rG4-seq (RT-stalling, K^+^ /Li^+^ ± PDS)	poly(A)	High (µg range)	In vitro, HeLa	3,845 (K^+^); 13,423 (K^+^ +PDS)
[21]	DMS, NAI probing + RT-stop	poly(A)	High	In vitro + in vivo, mESC	6,140 in vitro ~0% folded *in vivo*
[24]	G4RP-seq (BioTASQ pulldown)	poly(A)	High	In vivo, MCF7	>6,000 transcripts
[31]	SHALiPE-seq (NAI probing)	poly(A)	High	In vivo, Arabidopsis & rice	153 & 187
[27]	rG4-seq 2.0 (RT-stalling, low input)	poly(A)	Low (≥10 ng)	In vitro, HEK293T	1,364 at 10 ng
[28]	d-rG4-seq, chromatin RNA)	Chromatin-associated poly(A)	High	In vivo, mESC	54 *in vivo* vs. 5,538 in vitro (~1%)
[29]	ULI-rG4-seq (BG4 pulldown, low input)	Total RNA	Ultra-low (~100 oocytes)	In vivo, mouse oocytes	19,844 peaks (DMSO); 94,879 (cPDS)

rG4s are non-randomly distributed across mRNAs, showing significant enrichment in 5’and 3’ untranslated regions (UTRs) of mRNAs, intronic sequences near splice sites, and can also be found enriched on specific classes of non-coding RNAs, including ribosomal RNAs, transfer RNAs, and long non-coding RNAs [14,23,32–36].

## Nuclear rG4s: transient folding for gene regulation

Upon export from the nucleus, mature mRNAs lose their intronic sequences, altering their complement of regulatory elements and their structural landscape. In contrast to the cytoplasm, where mRNAs exist as mature transcripts, nuclear pre-mRNAs contain extensive intronic regions that provide additional sequence space for rG4 formation. This compartmental difference establishes distinct functional contexts in which nuclear rG4s can transiently fold during transcription and processing, whereas cytoplasmic rG4s must operate within the constraints of mature transcript architecture. RNA secondary structures form co-transcriptionally, with nascent transcripts able to pair within 25 bp of addition to the nascent chain [37]. Strong RNA structures near the polymerase can promote forward translocation of the polymerase but also modulate elongation speed depending on sequence context as shown for hairpins [38].

The precise folding kinetics of rG4s during transcription are not known; however, *in vitro* folding studies of individual rG4s suggest a timescale ranging from hundreds of milliseconds to seconds [39,40]. Given that 1.) Pol II elongation rates of ~0.5 kb per minute within the first few kilobases, to 2–5 kb per minute after ~15 kb and that co-transcriptional splicing takes an estimated 20–30 seconds [41], rG4s likely exhibit similar co-transcriptional and post-transcriptional effects on polymerase kinetics and RNA processing. Recent advances in rG4 mapping provide further circumstantial support for functional rG4 formation in pre-mRNA. Whole-cell rG4 sequencing in mouse oocytes using ULI-rG4-seq identified 14.74% of rG4 peaks mapping to intronic coordinates [29]. Given that 1.) introns represent a small fraction of total cellular RNA at steady state due to rapid splicing kinetics 2.) rG4s are enriched in 5’and 3’ UTR, the disproportionate intronic rG4 signal (while also potentially reflecting retained introns in mature transcripts) is consistent with G4 structure formation in nascent or partially processed RNA.

Studying the evolutionary conservation of rG4 motifs within gene regions provides compelling evidence for their functional importance within precursor mRNAs (pre-mRNAs). Conserved rG4 motifs exhibit approximately 3-fold enrichment near splice junctions with pronounced asymmetry favouring the non-template strand [32]. Analysis across mammalian and avian genomes shows this rG4 enrichment near splice sites is restricted to higher vertebrates and absent in invertebrates and plants, indicating that rG4-mediated splicing regulation co-evolved with the expansion of alternative splicing in these lineages [32]. The restriction to vertebrates suggests nuclear rG4s provided evolutionary advantages through reversible regulation that does not require permanent structural changes in mature transcripts [32,42].

## rG4s in pre-mRNA splicing regulation

The importance of G-rich sequences in vertebrate intron splicing regulation has been recognized for decades [43]. G triplets distributed throughout small vertebrate introns were shown to enforce intron borders and regulate splice site selection, with each G triplet contributing additively to splicing efficiency and spliceosome assembly [43]. These early observations established that G-rich intronic elements play functional roles in pre-mRNA processing, though the structural basis for their regulatory activity remained unclear at the time. Nuclear rG4 function in RNA processing is further demonstrated by their role in mirtron biogenesis. Mirtrons are noncanonical microRNAs generated through splicing rather than Drosha processing, and genome-wide analysis reveals that over 60% of mammalian mirtrons harbour rG4 motifs at their 5′ arm – nearly three times the prevalence observed in canonical pre-miRNAs. Experimental disruption of these 5′-arm rG4s severely impairs spliceosome-mediated excision of mirtrons and blocks downstream miRNA maturation, while mutations outside the rG4 motif have no effect on processing. This rG4 enrichment is specific to vertebrate mirtrons and is completely absent in invertebrates and plants, indicating that rG4-dependent regulation of splicing-mediated miRNA biogenesis represents an evolutionarily recent mechanism that likely prevents inappropriate processing of splicing-derived hairpins by the canonical miRNA machinery [42].

Direct evidence on a global scale regarding the folding dynamics of pre-mRNA G-quadruplexes does not exist to date; however, several studies indicate that they may form and that their folding state regulates splicing. For example, early studies on the Fragile X Messenger Ribonucleoprotein (FMRP) indicated that it binds its own mRNA via a G4 forming sequence, suggesting a potential role as a splicing enhancer [44]. An rG4 within exon 3 of the β-Site amyloid precursor protein cleaving enzyme 1 (BACE1) mRNA regulates alternative splicing through heterogeneous nuclear ribonucleoprotein H (HNRNPH) [45]. Protein-centric approaches have also demonstrated that transiently formed rG4 structures are involved in alternative splicing regulation. It has been suggested that rG4 structures promote alternative splicing via HNRNPF, where these structures promote exon inclusion through direct interaction with HNRNPF, suggesting that rG4s fold transiently during pre-mRNA processing to recruit splicing regulators [46]. We note that our work, by contrast, has shown that HNRNPF prefers unstable/unfolded rG4s, as demonstrated by RNA-Bind-n-Seq experiments under Li+ consistent with a preference for linear RNA substrates observed in structural studies of its three quasi-RRMs with RNA [47–49]. Additional hnRNP family members are implicated in splicing regulation through rG4 interactions, such as HNRNPH1, which promotes exon exclusion by binding G-rich sequences and shifting their equilibrium towards an unfolded non-G4 state [50].

All the above indicate that these proteins may bind structured G4s, but favour a linear form of these sequences either by unfolding them or remaining in contact with the structure and preventing refolding once a helicase actively unwinds them. Nevertheless, these studies imply that rG4s that form during transcription participate in splicing regulation. The role of rG4s and particularly their folding states in splicing has also been tested indirectly using compounds that bind and stabilize their structure. Pharmacological stabilization of rG4 structures near alternative splice sites in the Bcl-X pre-mRNA shifts-splice site selection in a ligand-dependent manner, providing indirect evidence that rG4 formation can modulate splicing outcomes [51].

## Nuclear rG4-protein interactions in phase separation

While much of the field has focused on deterministic roles for rG4s such as direct splice site selection, rG4s may more broadly modulate nuclear gene expression by influencing the biophysical properties of the local RNA environment, including through liquid-liquid phase separation, as discussed below. We have previously found that nuclear PKM2 interacts with folded RNA G-quadruplex (rG4) structures in precursor mRNAs (pre-mRNAs). Nuclear PKM2 depletion specifically reduces expression of transcripts containing rG4s near splice sites, while nuclear PKM2 accumulation increases their expression. This represents an unexpected connection between cellular metabolism and RNA structure-dependent gene regulation, where nuclear localization of a glycolytic enzyme contributes to co-transcriptional control mechanisms [47]. When we overexpressed HNRNPF the same transcripts (collectively the rG4ome) were downregulated. The rG4ome represents a regulome whose functional output is not deterministic but likely context-dependent, shaped by the dynamic interplay between rG4 structure, RBP availability, and the broader nuclear environment.

Cumulative evidence thus supports a broader model where nuclear rG4s – beyond their well-established role in telomere maintenance – serve as regulatory elements controlling multiple steps of RNA processing, from transcription elongation to splicing to non-coding RNA maturation and can directly regulate nuclear enzyme activity such as PRC2 during X-chromosome inactivation [28,52]. The intrinsic properties of rG4s – their ability to fold transiently and multivalently recruit proteins – combined with increasing evidence that IDR-containing RBPs interact with these structures, point to phase separation mechanisms in rG4-mediated regulation. Splicing occurs within biomolecular condensates such as nuclear speckles, which concentrate IDR-containing RBPs and splicing machinery through liquid-liquid phase separation [53]. rG4 folding dynamics during transcription may regulate gene expression and splicing by modulating RBP recruitment to these condensates

Cells organize many biochemical processes in membraneless compartments, biomolecular condensates that form spontaneously when proteins with intrinsically disordered regions and nucleic acids undergo weak multivalent interactions driving liquid-liquid phase separation (LLPS) from the cytoplasm or nucleoplasm [54–56]. A plethora of chromatin-associated proteins displaying high intrinsic disorder preferentially bind structured rG4s [57], suggesting that transient rG4 folding creates dynamic interaction networks within nuclear condensates that restructure in response to transcriptional demands, concentrating enzymatic machinery to regulate transcription and RNA processing. This mechanism parallels the stress granule formation observed in cytoplasmic compartments, suggesting that rG4-mediated phase separation represents a conserved organizational principle across cellular compartments with distinct roles (Table 2).

**Table 2.** RNA G-quadruplex protein interaction participating in membraneless organelles. Summary of cellular compartments where rG4-protein interactions contribute to phase separation and function. Listed are the subcellular localization, representative rG4-binding proteins, known rG4-containing RNA substrates and biological functions.

	Organelle/Granule	rG4-binding proteins	rG4 RNA substrates	Function	Key References
NUCLEAR	Nucleoli	NCL, NONO/PSPC1 (fibril centres), ZFP106, NPM1, SPATS2L (DNAPTP6)	rRNA G4s	Ribosome biogenesis, rRNA processing	[36,58–64]
	Nuclear speckles	NCL, NONO, NPM, HNRNPF, NPM1, SRSF1, HNRNPA1, HNRNPH/F	MALAT1 rG4s, Pre-mRNA G4s?	Splicing regulation, protein recruitment	[36,45,47–50,53,65,66]
	Paraspeckles	NONO, SFPQ, PSPC1, FUS, NCL, TDP43	NEAT1 rG4s	mRNA retention, gene regulation	[36,56,66–69]
	Telomeres	HNRNPA1, FUS	TERRA repeats	Telomere maintenance	[69–71]
CYTOPLASMIC	Stress granules	DNAPTP6, G3BP1, TIA-1, FUS, TDP-43, HNRNPA1, HNRNPA2/B1, HNRNPH/F	Mark2 mRNA, 3&5’ UTR rG4-mRNAs	Translational repression, cytoprotection	[20,31,50,67,69,72–76,77–83].
	Neuronal RNA granules	FMRP, FUS, TDP43, HNRNPA2/B1	SC1 RNA, PSD-95 mRNA	mRNA transport, localized translation	[68,75,84,85]

Nuclear biomolecular condensates containing rG4s include paraspeckles, nuclear speckles, and nucleoli, which, among other mechanisms, assemble through rG4-mediated recruitment of LLPS-driving proteins (Table 2). The long non-coding RNAs NEAT1 and MALAT1 contain multiple rG4-forming sequences that recruit RNA-binding proteins such as non-POU domain-containing octamer-binding protein (NONO), nucleophosmin (NPM1), and nucleolin (NCL), proteins harbouring intrinsically disordered regions essential for phase separation [36,60,64,66]. Nucleoli form through LLPS to facilitate the massive rRNA biogenesis demands and are enriched in both rDNA and rRNA G-quadruplexes [61,63]. Notably, rG4s appear more abundant than DNA G-quadruplexes in nucleoli (which assemble through phase separation), as demonstrated by differential sensitivity to RNAse versus DNAse treatment using Thioflavin T and fluorescence lifetime imaging microscopy [58]. Unlike cytoplasmic stress granules, which form reversibly in response to acute stress, nuclear rG4-containing condensates function constitutively to facilitate RNA processing (Table 2). NPM1 is implicated in LLPS driven phenomena and binds both RNA and DNA G4s [60,64]. Nuclear rG4s and their involvement in RNA processing through LLPS mechanism likely emerged in higher vertebrates alongside increased transcriptome complexity and alternative splicing diversity, unlike in the cytoplasm where they act as environmental sensors.

## Cytoplasmic rG4s

Unlike nuclear pre-mRNAs, where rG4s can exploit transient folding during transcription and splicing for gene regulation on single molecules, cytoplasmic mature mRNAs cannot ensure removal of transient rG4s by splicing out intronic sequence space, but rather have to rely on accessory factors, such as RBPs and helicases, to change rG4 folding status, complicating their use as regulatory elements. In this environment, rG4 folding appears to serve different functions, primarily related to translation, stress response and mRNA stability [86].

A study including 15,000 whole-genome sequences showed that the level of negative selection pressure acting on central guanines of putative rG4s in UTRs is similar to that acting on missense variants in protein-coding sequences suggesting a functional role for, most likely folded, rG4s within UTRs of mature mRNA [87]. RNA G-quadruplexes in 5’ UTRs regulate translation through multiple mechanisms, primarily through ribosome scanning impedance [88]. Early work demonstrated that a highly conserved rG4 in the NRAS 5“ UTR represses translation, with similar effects observed for ZIC1 mRNA [89,90]. Ribosomes can overcome rG4 barriers during scanning but at reduced efficiency, resulting in translational suppression [91]. Although there is a general trend for suppression of translation by 5” UTR rG4 as shown by functional assays, rG4s can be essential for IRES-mediated translation initiation [92]. In addition, rG4s in 5’ UTRs recruit the eIF4A helicase to enable preferential translation of growth-promoting oncogene mRNAs in cancer cells [93].

Nevertheless, recent high-throughput approaches have revealed a more complex picture of rG4 behaviour in cytoplasmic compartments. High-throughput DMS-seq analysis demonstrated that rG4s within poly(A)-selected RNA remain globally unfolded in eukaryotic cells under normal cell culture conditions [21]. Thousands of RNA regions capable of forming stable rG4s *in vitro* exist in unfolded conformations within cells. This constitutive unfolding is an active cellular process that requires dedicated machinery, including helicases, RNA-binding proteins, and ATP-dependent remodelling complexes. This research establishes that rG4s within mature RNAs are strictly unfolded in mESCs and HEK293T under conditions that favour cell survival and proliferation in culture.

## rG4s and stress granule assembly

However, rG4s in mature mRNAs may fold under stress conditions, and it seems that, unlike in the nucleus, where the dynamics of unfolding pose a regulatory layer, in the cytoplasm, stress-induced rG4 formation is at the core of a cellular response [86]. Stress granules (SGs) are dynamic ribonucleoprotein assemblies that form during cellular stress to coordinate mRNA fate and translation control [94,95]. These membraneless organelles concentrate stalled translation initiation complexes, specific mRNAs, and regulatory proteins through liquid-liquid phase separation mechanisms driven by multivalent low-affinity interactions between intrinsically disordered protein regions and RNA [96,97]. The formation and composition of SGs depend critically on RNA-protein and RNA-RNA interactions, in which RNA has been proposed to act as nucleation sites for ribonucleoprotein complex formation [98]. SG formation enables rapid cellular adaptation to stress by compartmentalizing the translation machinery and storing mRNA. The concentration of specific mRNAs and regulatory factors within stress granules facilitates stress response while protecting essential transcripts from degradation [98–101].

Among RNA elements that contribute to SG nucleation, rG4s have emerged as key players. Early evidence came from tRNA-derived stress-induced RNAs (tiRNAs), which assemble into uniquely stable tetramolecular rG4 structures consisting of five G-tetrad layers, a structurally distinct class from the intramolecular rG4s found in mRNAs [73,102]. Disruption of these rG4 structures abolishes tiRNA-mediated stress granule formation in vivo, establishing rG4 assembly as causally required for this stress response pathway [102]. Consistent with this, DNAPTP6 coordinates neuronal stress granule assembly in an rG4-dependent manner [78] and G3BP1 binds to rG4s in a stress-dependent manner to stabilize mRNAs in stress granules [80]. Several studies have therefore suggested that rG4-protein interactions broadly contribute to stress granule formation [86], though whether folded rG4 structures generally drive this process or form as byproducts of stress granule assembly remains unclear. DHX36 (also known as RHAU), which prevents the accumulation of translationally inactive mRNAs with G4s in UTRs, illustrates this complexity [76]. DHX36 was first shown to localize to stress granules in 2008 through its unique N-terminal RNA-binding domain [103] and subsequent characterization of its unwinding activity on DNA and RNA G4s directly linked stress granules with rG4s [104,105]. Yet, DHX36 depletion leads to increased rG4 formation and altered mRNA stability, particularly under stress conditions where helicase activity may be compromised [86], while other factors including DDX3X (unwinds) and CNBP (prevents folding), similarly maintain rG4s in unfolded states, suggesting that rG4 folding must be actively suppressed under normal conditions [106,107]. Recent work has, however, shown that DHX36 involvement in stress granule assembly following arsenite treatment is independent of mRNAs bearing G-quadruplex motifs [79], suggesting that rG4 formation during stress may be a consequence of helicase displacement from mRNAs rather than a primary nucleating event. Yet, regardless of whether rG4 folding drives or follows stress granule assembly, stress-induced rG4 formation has clear functional consequences for mRNA fate.

## Stress-induced rG4 folding and mRNA stability

A recent study has shown that stress-induced cytoplasmic rG4 folding preferentially occurs within mRNA 3’ UTRs, where folded structures enhance transcript stability [81]. Using DMS-seq combined with rG4-specific pull-down approaches, the researchers identified hundreds of endogenous stress-responsive rG4. Various stresses, including starvation, oxidative stress (sodium arsenite), and thermal stress (cold conditions), induce rG4 folding, each producing an approximately 2-fold increase in BG4 immunofluorescence. Critically, this folding is reversible upon stress removal and requires ATP for the unfolding process, indicating active regulation of the folded-unfolded equilibrium.

These findings suggest that different stress response mechanisms may eventually lead to rG4 folding in cells. All these stresses converge on the integrated stress response (ISR) pathway, which involves eIF2α phosphorylation and global translation attenuation [108]. The lack of active transcription and reduced translation during ISR activation may play a crucial role in rG4 formation, as decreased helicase occupancy on mRNAs could permit structure formation. mRNAs containing 3’ UTR rG4 sequences exhibit significantly increased stability under starvation conditions, with similar protective effects observed upon treatment with rG4-stabilizing ligands, including cPDS and BRACO-19. This represents a cytoprotective mechanism in which normally unfolded cytoplasmic sequences adopt folded conformations that shield mRNAs from the degradation machinery. The 3’ UTR localization suggests specific targeting of post-transcriptional regulatory networks that control mRNA decay pathways [81]. These observations suggest that cytoplasmic rG4 dynamics constitute a threshold-dependent stress response system that fundamentally diverges from nuclear rG4-mediated gene regulation.

Extreme stresses such as arsenite induce rapid eIF2α phosphorylation-dependent polysome collapse, releasing mRNAs for stress granule assembly [99,101,109–112]. Under physiological conditions, however, cytoplasmic G4 foci exist in the adult mouse hippocampus without any exogenous stress, and rG4 folding drives neuroprotective stress granule assembly through DNAPTP6 during oxidative stress [77,78]. Supporting a direct role for rG4 structure in this process, self-assembly of rG4s in vitro was shown to boost DNAPTP6 phase separation, suggesting that rG4 folding itself is sufficient to nucleate stress granules rather than merely accompanying their assembly.

These studies suggest that rG4 dynamics play a more significant regulatory role under physiological conditions, in which cells may maintain partial function rather than undergo an acute crisis. Consistent with this view, in plants, cold stress triggers rG4 folding in specific mRNAs, enhancing their stability and maintaining translation of cold-responsive genes, a temperature-dependent mechanism that operates well within physiological bounds [25]. The challenge of recreating such conditions in the laboratory becomes apparent when considering that standard cell culture conditions lack genuine physiological stress, raising questions about whether acute stress protocols faithfully reproduce the rG4 dynamics occurring *in vivo*.

**Figure 2.**
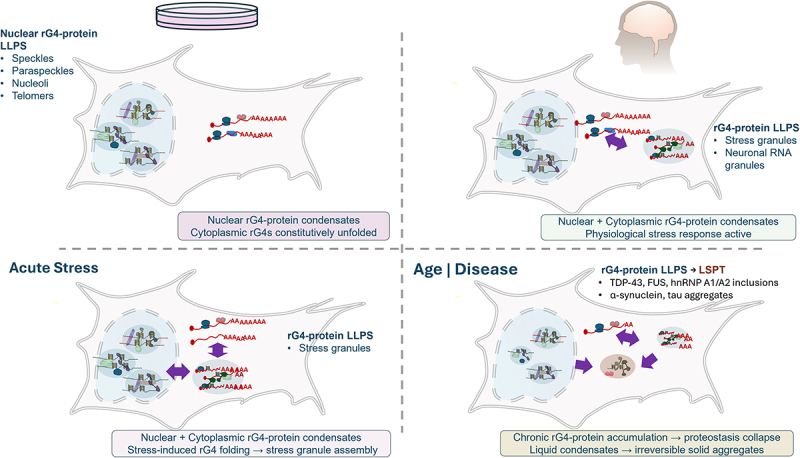
RNA G-quadruplexes exist in two functionally distinct worlds shaped by cellular compartment and stress. Schematic comparison of rG4 behaviour in cell culture (left) versus in vivo brain (right) under normal (top) and stress/disease (bottom) conditions. In standard cell culture under normal conditions (top left), only nuclear rG4 condensates are present; cytoplasmic rG4s are constitutively maintained in an unfolded state by helicases (DHX36, DDX3X) and RNA-binding proteins (CNBP). In vivo (healthy brain, top right), both nuclear and cytoplasmic rG4 condensates coexist: nuclear rG4s regulate co-transcriptional RNA processing while cytoplasmic rG4 condensates participate in physiological stress responses. Under acute stress in culture (bottom left), integrated stress response (ISR) activation and eIF2α phosphorylation reduce helicase activity, permitting cytoplasmic rG4 folding and stress granule assembly. In aged or neurodegenerative brain (bottom right), chronic rG4 accumulation drives pathological liquid-to-solid phase transitions, converting protective condensates into irreversible aggregates containing α-synuclein, tau, TDP-43, and FUS.

Supporting this concern, prolonged low-dose stress preconditioning – which may better approximate the mild physiological stresses experienced by cells *in vivo*, paradoxically prevents stress granule formation in response to subsequent acute stress [113]. This suggests that standard acute stress protocols used to study stress granules *in vitro* (e.g. high-dose arsenite treatment of unstressed cells) may not accurately recapitulate the stress granule dynamics occurring in organisms that experience baseline stress levels. Consistent with this, acute versus chronic loss of DHX36 leads to opposing effects on rG4 levels in oocytes, suggesting that cells activate compensatory mechanisms to maintain rG4 structural homoeostasis [29]. The major question that the above findings raise is whether the various conditions cells are constantly exposed to *in vivo* impact the folding/unfolding balance and whether cellular models can even recapitulate this phenomenon (Figure 2). Notably, most cell culture conditions far exceed the physiological nutrients cells are supplied *in vivo*, such as oxygen, glucose, pyruvate, and amino acids [114–117]. Not only are cells exposed to much lower nutrient levels *in vivo*, but they are often under acute, chronic, or age-related intrinsic and extrinsic stresses [118]. Longevity is intimately related to the organism’s ability to effectively cope with stress [119]. Loss of this ability is particularly important to long-lived cells *in vivo*, such as neurons, rendering chronic and age-related stresses drivers of neurodegeneration, where rG4-protein interactions emerge as key players and provide insights into the function of rG4-protein interactions in health and disease [120–123].

## rG4-protein interactions in neurodegeneration

Until recently, the involvement of rG4s in neurodegeneration was largely speculative. Over the past few years, however, a coherent picture has emerged: rG4s are not passive bystanders but active participants in neurodegeneration, functioning as protective scaffolds during acute stress and as pathological drivers when their homeostasis fails. The first indication that rG4s play physiological roles in neurons came from the detection of cytoplasmic G4 foci by BG4 immunostaining in adult mouse hippocampal CA1 region under physiological conditions, establishing that rG4 formation occurs in vivo in healthy neurons (Figure 2). This observation is functionally significant: in cultured Neuro-2A cells, stress-induced rG4 folding nucleates neuroprotective stress granules through the multivalent RNA-binding protein DNAPTP6 (SPATS2L/SGNP), which coordinates phase separation during oxidative stress. DNAPTP6-positive granules sequester translationally stalled mRNAs together with G3BP1 and FMRP, and DNAPTP6 knockdown abolishes this response, causing synaptic dysfunction and neuronal death [78]. G3BP1 binds rG4 directly through its RGG domain to modulate mRNA stability during stress [80], while FMRP colocalizes with rG4-containing stress granules via RGG motifs that recognize rG4s [72,85].

The mechanistic basis for these protective interactions is increasingly understood [124]. Both DNA and RNA G4s function as ATP-independent chaperones that prevent toxic protein aggregation and may even catalyse protein folding as foldases, an activity previously considered exclusive to ATP-dependent chaperonins [125–130]. This chaperone activity, combined with the multivalent protein-recruiting properties of G4 structures, explains why rG4s are so effective at nucleating the reversible phase-separated condensates that protect neurons during acute stress [131]. The same properties that make rG4s protective become liabilities when homeostasis breaks down. The proteins that normally engage rG4s in functional condensates, TDP-43, FUS, HNRNPA1, HNRNPA2B1, and NCL, are among the most prominent ALS and FTD pathology proteins, and in each case, the disease mechanism involves a failure of their normal rG4-dependent nuclear function followed by cytoplasmic mislocalization and aggregation. TDP-43 is a predominantly nuclear protein that binds both GU-rich sequences and rG4 structures to regulate splicing, mRNA stability, and transport; rG4 stabilization with small molecules reduces stress-induced TDP-43 condensation and toxicity, directly demonstrating that rG4-TDP-43 interactions maintain proteostasis under stress [82]. In ~97% of ALS cases, TDP-43 is depleted from the nucleus and accumulates in detergent-insoluble cytoplasmic inclusions, and ALS mutations in its glycine-rich region specifically impair rG4 binding and RRM recruitment, disrupting mRNA transport [67,68,83,132]. FUS follows the same logic: a nuclear G4-binding protein involved in transcription, splicing, and DNA repair, whose ALS mutations disrupt nuclear localization, rG4 binding, and LLPS dynamics, driving cytoplasmic mislocalization and aggregation [67,69,133]. HNRNPA1 and HNRNPA2B1 are nuclear splicing regulators that recognize G-quadruplexes through RGG domains; ALS mutations in their prion-like domains and nuclear localization signals cause cytoplasmic amyloid fibril formation and loss of nuclear function [70,71,74,75]. NCL, a nucleolar rG4-binding protein essential for ribosome biogenesis, is sequestered by the G-quadruplex-forming C9orf72 (GGGGCC)n repeat expansions, causing nucleolar stress and ribosomal dysfunction in ALS/FTD [62]. The pattern is consistent: disease mutations disrupt normal nuclear rG4-protein interactions while simultaneously promoting aberrant cytoplasmic aggregation, suggesting a collapse of the homoeostatic balance that healthy neurons depend on. This is further exemplified by diseases driven by pathological repeat expansions, where transcripts form stable, repetitive G4 structures that actively sequester rG4-binding proteins away from their normal functions. In Fragile X syndrome, CGG repeat expansions in the *FMR1* 5’ UTR form rG4 structures that simultaneously suppress FMRP translation and sequester FMRP protein through RGG domain binding, creating a self-reinforcing pathological loop [84,85]. In C9orf72 ALS/FTD, GGGGCC repeat-containing RNA foci accumulate in both nuclear and cytoplasmic compartments of patient neurons, sequestering NCL and HNRNPH. The repeat RNA also undergoes RAN translation to produce toxic dipeptide repeat proteins and recently Zfp106 was found to inhibit both RNA foci accumulation and RAN translation [59,62,134–139]. rG4 accumulation also emerges as a feature of sporadic neurodegeneration. In synucleinopathies, rG4s act as scaffolds for α-synuclein aggregation: purified α-synuclein binds rG4s directly through its N-terminus, rG4s undergo Ca^2 +^ -induced phase separation that accelerates α-synuclein sol-gel transition, and forced rG4 assembly via optogenetics is sufficient to trigger α-synuclein aggregation and neuronal dysfunction *in vivo* [140]. In Alzheimer’s disease, BG4 immunostaining of post-mortem human hippocampus reveals that rG4 accumulation increases with both age and AD severity, with the largest increases in the outer molecular layer and CA4 hilar region, areas of prominent early synaptic loss [141].

Consistent with this, rG4 structures promote tau aggregation both in cellular seeding assays using G4 sequences isolated from human brain aggregates and in Ca^2 +^ -driven reconstitution experiments with purified components, suggesting that rG4-tau interactions may contribute to AD pathology [141,142]. Intriguingly, PKM2, which we found binds rG4s in the nucleus, forms aggregates in aged mouse brain and liver, raising the question of whether age-related rG4 accumulation contributes to PKM2 aggregation and the associated decline in aerobic glycolysis [47,143,144]. Collectively, these findings converge on a unified model: in healthy neurons, nuclear rG4-protein interactions regulate RNA processing while cytoplasmic rG4s remain suppressed until stress triggers their protective phase separation. When this compartmental balance fails through disease mutations, chronic stress, or ageing, protective condensates convert into irreversible aggregates, driving neurodegeneration. The dual nature of rG4s as both neuroprotective chaperones and pathological aggregation scaffolds is exemplified by therapeutic approaches targeting opposite ends of the same axis: small molecule stabilization of rG4 structures reduces TDP-43 condensation and toxicity in cellular models, consistent with their proposed chaperone activity [82], while inhibition of rG4 phase separation with 5-aminolevulinic acid attenuates α-synuclein aggregation and motor deficits in synucleinopathic mice [140]. Together, these findings suggest that the therapeutic goal is not simply promoting or inhibiting rG4 folding, but correcting the specific failure mode in each disease context.

## Conclusions

Nuclear rG4s fold transiently during transcription to orchestrate RNA processing, while cytoplasmic rG4s stay unfolded to maintain translation but fold under stress to coordinate protective responses. Despite these distinct functional roles, nuclear and cytoplasmic rG4s share common biophysical properties: both interact with IDR-containing proteins and both can nucleate phase-separated condensates. The difference lies in timing and regulation: nuclear rG4s fold co-transcriptionally to recruit splicing factors, while cytoplasmic rG4s remain suppressed until stress triggers their folding. These functions emerged through different evolutionary paths. Nuclear rG4 regulation of splicing arose specifically in vertebrates: rG4 motifs show ~3-fold enrichment near splice junctions with strand asymmetry, a pattern absent in invertebrates and plants [32]. Over 60% of mammalian mirtrons contain 5′-arm rG4s that promote splicing, whereas plant and invertebrate mirtrons completely lack this enrichment [42]. In contrast, cytoplasmic stress-responsive rG4 folding represents an ancient mechanism conserved across kingdoms, from human cells responding to oxidative stress to plants using rG4 folding for cold adaptation [81,145]. Nuclear rG4s evolved in vertebrates to meet complex RNA processing demands; cytoplasmic rG4s remained ancestral stress sensors. Despite their distinct compartmental roles, nuclear and cytoplasmic rG4s share a common mechanistic principle: multivalent recruitment of IDR-containing proteins to drive liquid-liquid phase separation, whether to organize constitutive nuclear condensates for RNA processing or to nucleate transient cytoplasmic stress granules for mRNA protection.

The plethora of studies on neurodegeneration reveals the pathological metamorphosis of this cytoplasmic system. Under acute stress, rG4-protein interactions organize reversible phase separation that protects cells by sequestering mRNAs and scaffolding protective complexes. But when stress becomes chronic or rG4-binding protein homoeostasis fails, the same interactions drive irreversible aggregation. Recent work on lithium raises the possibility, albeit speculative, that ionic homoeostasis may influence what tips the balance towards pathological rG4 states. Endogenous lithium is significantly reduced in individuals with mild cognitive impairment, with Li bioavailability further reduced in AD by amyloid sequestration, and dietary lithium restriction in mice markedly increased amyloid-β and phospho-tau pathology while accelerating cognitive decline [146]. Whether changes in lithium levels observed among healthy individuals and AD patients occur at concentrations sufficient to influence rG4 dynamics (unlike potassium, lithium levels used *in vitro* for experimentation are usually a magnitude or two higher than *in vivo*) remains unknown. However, recent kinetic studies show that even at concentrations far below potassium (10 mM Li+ with 150 mM K+), lithium accelerates potassium-driven G4 folding rates by up to 8-fold, suggesting physiological lithium levels may modulate rG4 dynamics in ways not previously appreciated [147]. Targeting the transition from reversible to pathological rG4 states offers therapeutic opportunities for restoring proteostasis in ageing and neurodegeneration.
